# The prognostic value of optic nerve sheath diameter in patients with subarachnoid hemorrhage

**DOI:** 10.1186/s13054-019-2360-6

**Published:** 2019-02-26

**Authors:** Sangkil Lee, Yong Oh Kim, Ji Sun Baek, Jeong-Am Ryu

**Affiliations:** 1 Department of Neurology, ChungBuk National University Hospital, Cheongju, Republic of Korea; 2 Department of Critical Care Medicine, Samsung Medical Center, Sungkyunkwan University School of Medicine, 81 Irwon-ro, Gangnam-gu, Seoul, 06351 Republic of Korea; 3 Department of Ophthalmology, Konyang University, Kim’s Eye Hospital, Myung-Gok Eye Research Institute, Seoul, Republic of Korea; 4 Department of Neurosurgery, Samsung Medical Center, Sungkyunkwan University School of Medicine, 81 Irwon-ro, Gangnam-gu, Seoul, 06351 Republic of Korea

**Keywords:** Optic nerve sheath diameter, Brain computed tomography, Subarachnoid hemorrhage

## Abstract

**Background:**

We evaluated the role of optic nerve sheath diameter (ONSD) using brain computed tomography (CT) in predicting neurological outcomes of patients with subarachnoid hemorrhage (SAH).

**Methods:**

This was a retrospective, multicenter, observational study of adult patients with SAH admitted between January 2012 and June 2017. Initial brain CT was performed within 12 h from onset of SAH, and follow-up brain CT was performed within 24 h from treatment of a ruptured aneurysm. Primary outcome was neurological status at 6-month follow-up assessed with the Glasgow Outcome Scale (GOS, 1 to 5).

**Results:**

Among 223 SAH patients, 202 (90.6%) survived until discharge. Of these survivors, 186 (83.4%) manifested favorable neurological outcomes (GOS of 3, 4, or 5). In this study, the ONSDs in the group of patients with poor neurological outcome were significantly greater than those in the favorable neurological outcome group (all *p* < 0.01). Intracranial pressure (ICP) was monitored in 21 (9.4%) patients during the follow-up CT. A linear correlation existed between the average ONSD and ICP in simple correlation analysis (*r* = 0.525, *p* = 0.036). Analysis of the receiver  operating characteristic curve for prediction of poor neurological outcome showed that ONSD had considerable predictive value (*C*-statistics, 0.735 to 0.812). In addition, the performance of a composite of Hunt and Hess grade and ONSD was increasingly associated with poor neurological outcomes than the use of each marker alone.

**Conclusions:**

ONSD measured with CT may be used in combination with clinical grading scales to improve prognostic accuracy in SAH patients.

## Key messages

- In this study, patients with poor-grade SAH showed considerable survival (79.5%) and favorable neurological prognoses (61.4%).
- Concurrent measurement of ONSDs using brain CT and ICP was moderately correlated.
- The ONSDs of patients with poor neurological outcome were significantly greater than those with a favorable neurological outcome.
- A composite parameter of H-H grade and ONSD measured with CT may increase the accuracy of prognosis in patients diagnosed with SAH (H-H grade > 3 and follow-up ONSD_index_ > 6.22).

## Background

Subarachnoid hemorrhage (SAH) is a complex neurovascular syndrome and a devastating condition associated with high mortality and morbidity rates for those who survive the initial hemorrhage [1–3]. In SAH patients, the level of consciousness at admission is the most critical early predictor of clinical outcome [1, 4]. Patients with poor-grade SAH (grades 4 to 5) based on Hunt and Hess [5] or World Federation of Neurosurgical Societies (WFNS) grading scales [6] show high mortality and poor neurological outcomes [7]. However, recent reports suggest that patients with poor-grade SAH treated early and aggressively with coil embolization and neurointensive care achieved favorable neurological outcomes [3, 7]. Therefore, new predictors other than the initial level of consciousness are needed to accurately evaluate the prognosis of these patients.

Poor neurological outcomes of SAH patients are usually secondary to early brain injury, rebleeding, or delayed cerebral ischemia [1, 8, 9]. Early brain injury is associated with intracranial hypertension [1, 9, 10]. Therefore, early monitoring of intracranial hypertension may allow prediction of neurological outcomes in these patients. Optic nerve sheath diameter (ONSD) has been proposed as an alternative measure for the detection of intracranial hypertension [11, 12]. The ONSD may be associated with neurological outcomes of SAH patients. However, it has not been reported whether ONSD may facilitate systemic evaluation of neurological outcomes of patients with SAH. Therefore, the objective of this study is to investigate if ONSD with some modifications could be used to predict neurological outcomes of patients with SAH.

## Methods

### Study population and design

**Fig. 1:**
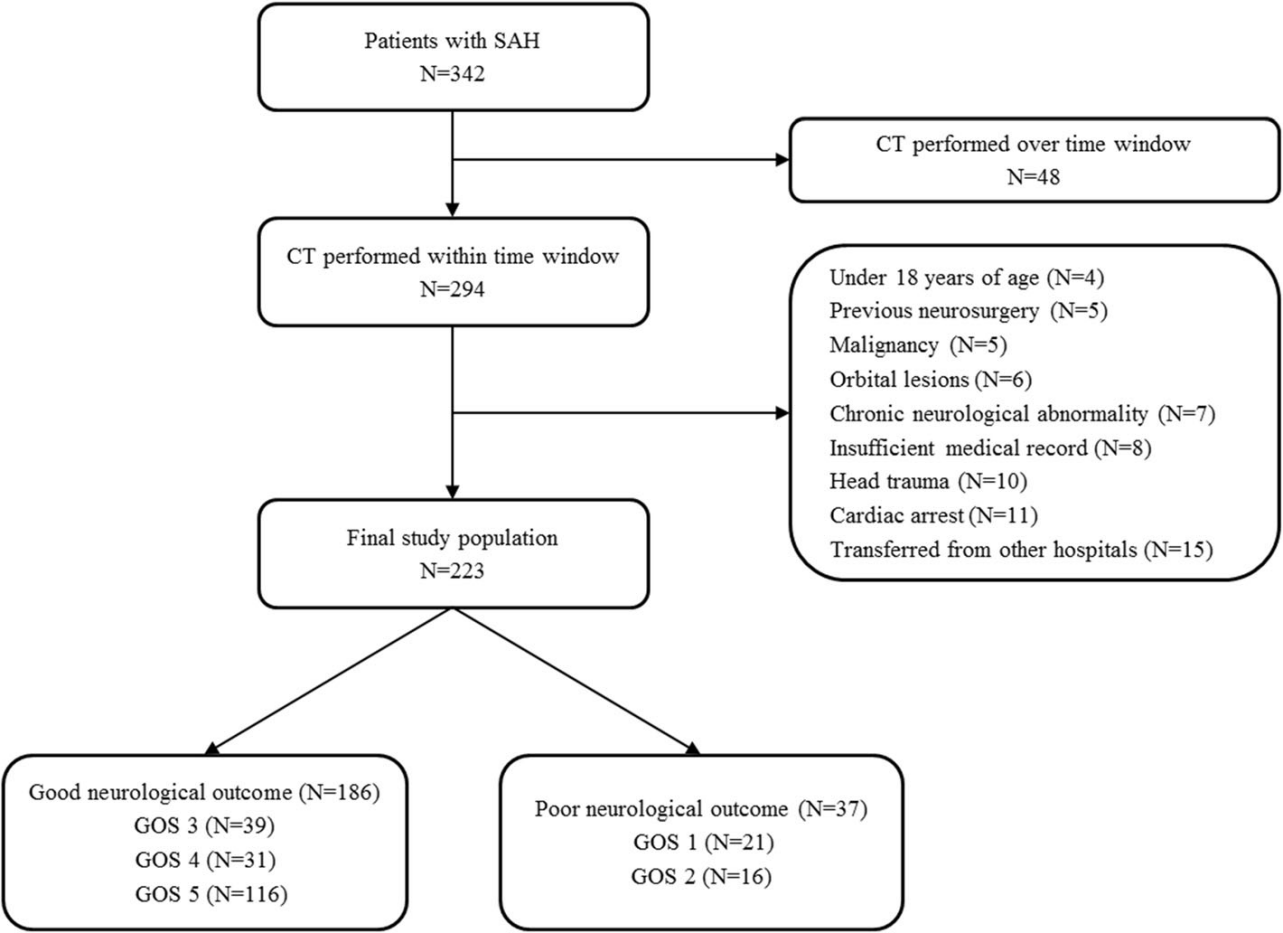
Study flow chart. SAH subarachnoid hemorrhage, CT computed tomography, GOS Glasgow Outcome Scale

This was a retrospective, multicenter, observational study of adult patients with SAH admitted to the neurosurgical intensive care unit at ChungBuk National University Hospital (CBNUH) and Samsung Medical Center (SMC) between January 2012 and June 2017. The study was approved by the institutional review boards of CBNUH (CBNUH 2018-08-014-001) and SMC (SMC 2018-07-154). The requirement for informed consent was waived due to the retrospective study design. We included patients with (1) SAH admitted to the neurosurgical intensive care unit during the study period, (2) brain computed tomography (CT) within 12 h from the onset of SAH, (3) follow-up brain CT within 24 h of treatment for ruptured aneurysm, and (4) follow-up brain CT within 48 h from the onset of SAH if they were not treated for aneurysm. Of these patients, we excluded patients (1) under age 18; (2) with malignancy and whose expected life span was less than 1 year; (3) with insufficient medical records; (4) with a history of head trauma, neurosurgery, cardiac arrest, or chronic neurological abnormality on admission; (5) transferred from other hospitals after more than 24 h of SAH onset; or (6) with orbital anomaly, orbital mass lesions, and ocular or retro-orbital injury. We analyzed a total of 223 patients diagnosed with SAH in this study (Fig. 1).

### Measurements and outcomes

**Fig. 2:**
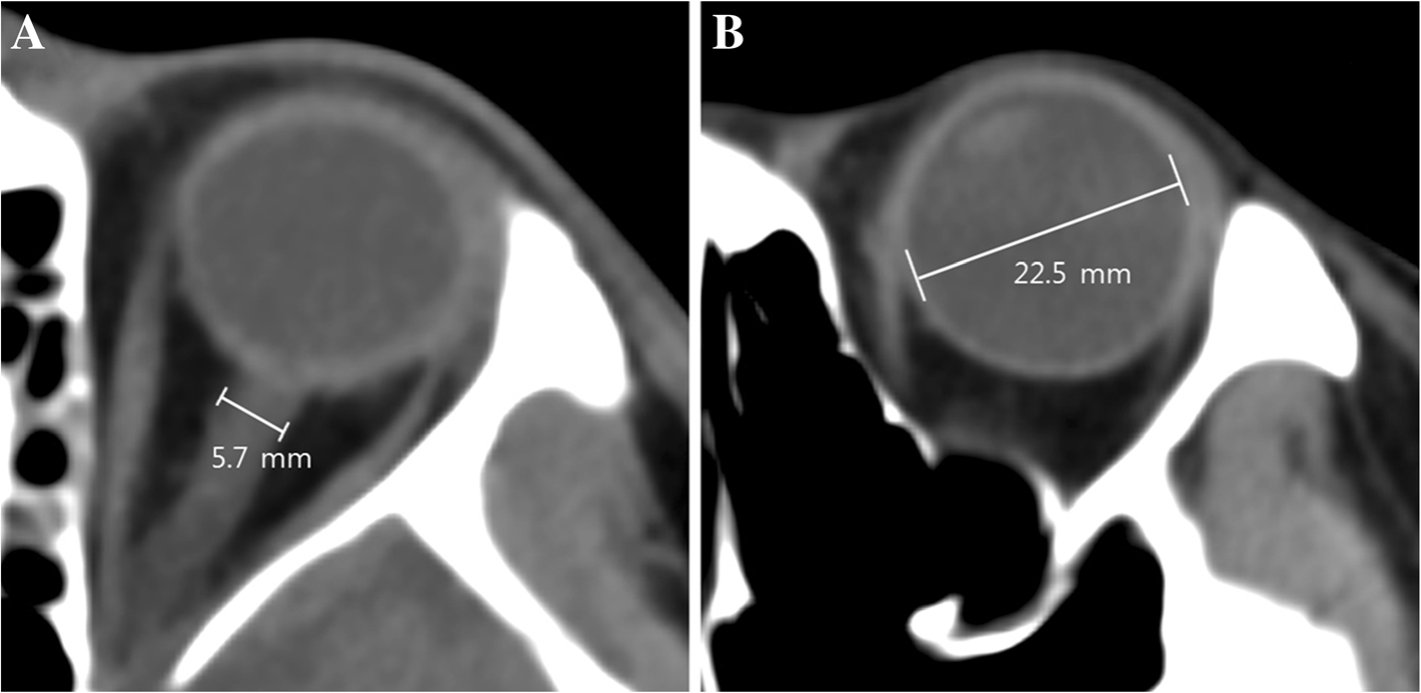
Measurement of optic nerve sheath diameter (**a**) and eyeball transverse diameter (**b**) on the brain computed tomography scan

In this study, we defined poor-grade SAH as grades 4 to 5 according to Hunt and Hess (H-H) [1, 4, 5]. The primary outcome was neurological status 6 months later assessed with a Glasgow Outcome Scale (GOS, 1 to 5) [12]. Patients with GOS scores of 3, 4, and 5 indicated favorable neurological outcomes whereas GOS scores of 1 and 2 indicated poor neurological outcomes. We thoroughly reviewed medical records, and two independent neurologists measured the patients’ GOS scores. Initial brain CT angiography and follow-up CT were performed within a specified time window. All the CT studies were performed using 64-channel scanners (Brilliance 64, Philips Medical Systems, Best, the Netherlands, at CBNUH and Light Speed VCT, GE Healthcare, Milwaukee, WI, USA, at SMC) with a 5-mm-slice width. Brain CT images were reviewed by two independent neurologists (SL and JAR). Investigators who were blinded to clinical information evaluated each of the patients’ CT scans using commercial image-viewing software (Picture Archiving and Communication System; Maroview 5.3 Infinitt Co., Seoul, Republic of Korea, at CBNUH and Centricity RA1000 PACS Viewer, GE Healthcare at SMC). The ONSD and eyeball transverse diameter (ETD) were measured using the same initial CT and subsequent scans. The ONSD was measured at a distance of 3 mm behind the eyeball, immediately below the sclera in a perpendicular vector with reference to the linear axis of the nerve (Fig. 2a) [11–13]. The images were changed to the “chest/abdomen” window (window width 300 and window level 10) and magnified fourfold on the particular image slice that demonstrated the largest diameter of the optic nerve sheath [12]. The ONSD was measured from one side of the optic nerve sheath to the other as a section through the center of the optic nerve [13]. The transverse diameter of the eyeball was chosen because the ONSD is usually measured in the transverse plane [14]. ETD was defined as the maximal transverse diameter of the eyeball measured from one side of the retina to the other (in-to-in, Fig. 2b) [14, 15]. The ONSD_average_ and the ETD_average_ measured for the patient’s left and right eyes were averaged to yield a mean value. The ONSD_index_ was defined as median ETD (22.7 mm) multiplied by the average value of bilateral ONSDs divided by the average value of bilateral ETDs (22.7 × ONSD_average_/ETD_average_). The intracranial pressure (ICP) at the time of follow-up CT was designated as the immediate ICP after the CT scan.

### Statistical analyses

Clinical and laboratory data were collected by a trained study coordinator using a standardized case report form. All data are presented as means ± standard deviations (SD) for continuous variables and numbers (percentages) for categorical variables. We compared data using the Student’s *t*-test for continuous variables and chi-square test or Fisher’s exact test for categorical variables. A scatter plot was drawn to ascertain the relationship between simultaneously measured ONSD and ICP. We calculated Pearson’s correlation coefficient (*r*) to evaluate the correlation between ONSD and ICP. We assessed the predictive performance of ONSDs and ONSD indices using the areas under the curve (AUCs) of the receiver operating characteristic (ROC) curves for sensitivity vs. 1-specificity. We compared AUCs using the nonparametric approach published by DeLong et al. [16] for two correlated AUCs. We obtained optimal cutoffs for each ONSD and their modifications to predict poor neurological outcomes by ROC curve and Youden index [17, 18]. We used the bidirectional elimination technique in the stepwise selection method to select clinically and statistically meaningful predictor variables. The adequacy of the prediction model was also determined using the Osius and Rojek test of goodness-of-fit [19]. All tests were two-sided, and *p* < 0.05 was statistically significant. We analyzed the data using IBM SPSS version 20 (IBM, Armonk, NY, USA).

## Results

### Baseline characteristics and clinical outcomes

The mean age of patients was 58.7 ± 13.0 years, and 84 (37.7%) patients were males. Hypertension (39.5%) and smoking (26.9%) were the most common comorbidities among the SAH patients. Eighty-three (37.2%) patients were H-H grades 4 or 5, and 96 (43.0%) were WFNS grades 4 or 5. WFNS grade was higher in patients included under the poor neurological outcome group compared with the favorable neurological outcome group (4.4 ± 1.1 vs. 2.4 ± 1.5, *p* < 0.001). Fisher grade and modified Fisher grade were also higher in the poor neurological outcome group compared with favorable neurological outcome group (3.9 ± 0.3 vs. 3.2 ± 0.7, *p* < 0.001 and 3.8 ± 0.4 vs. 2.9 ± 1.0, *p* < 0.001, respectively). Baseline characteristics of SAH patients are presented in Table 1. Anterior communicating artery (29.6%) and middle cerebral artery (25.1%) were the most common locations of a ruptured aneurysm. However, no aneurysm or ruptured aneurysm was detected in 14 patients (6.3%). Ruptured aneurysm was treated within 72 h in most patients (88.8%). Endovascular coiling of the ruptured aneurysm was performed in 124 (55.6%) patients, and surgical clipping was performed in 75 (33.6%) patients; delayed cerebral ischemia was observed in 46 (20.6%) patients. Treatment characteristics of SAH patients are presented in Table 2. Two hundred two patients (90.6%) survived to discharge. Of these 202 survivors, 186 (83.4%) had favorable neurological outcomes (GOS of 3, 4, or 5, Fig. 1). Among the 83 (37.2%) patients with poor-grade SAH, 66 survived to discharge (79.5%), and 51 (61.4%) had favorable neurological outcomes.

**Table 1:** Baseline characteristics

	Favorable neurological outcome (*n* = 186)	Poor neurological outcome (*n* = 37)	*p* value
Age (year)—mean ± SD	57.7 ± 12.1	63.8 ± 16.0	0.034
Gender, male—no. of patients (%)	69 (37.1%)	15 (40.5%)	0.834
BMI (kg/m^2^)—mean ± SD	23.7 ± 3.8	22.8 ± 3.5	0.219
Comorbidities—no. of patients (%)			
Hypertension	73 (39.2)	15 (40.5)	0.999
Current smoker	51 (27.4)	9 (24.3)	0.853
Diabetes mellitus	10 (5.4)	7 (18.9)	0.013
Dyslipidemia	10 (5.4)	4 (10.8)	0.382
Previous TIA or stroke	6 (3.2)	5 (13.5)	0.026
Ischemic heart disease	6 (3.2)	4 (10.8)	0.109
Malignancy	6 (3.2)	2 (5.4)	0.867
Chronic kidney disease	4 (2.2)	3 (8.1)	0.167
The interval from symptom onset to initial CT	4.5 ± 5.9	2.7 ± 3.4	0.015
The interval from symptom onset to follow-up CT	26.5 ± 13.4	24.5 ± 13.4	0.385
The interval from initial CT to follow-up CT	22.0 ± 12.1	21.7 ± 13.7	0.876
Hunt and Hess classification—no. of patients (%)			< 0.001
2	98 (52.7)	2 (5.4)	
3	37 (19.9)	3 (8.1)	
4	29 (15.6)	7 (18.9)	
5	12.1 ± 3.9	6.4 ± 3.6	
Modified Fisher classification—no. of patients (%)			< 0.001
1	29 (15.6)	0 (0)	
2	10 (5.4)	0 (0)	
3	101 (54.3)	9 (24.3)	
4	46 (24.7)	28 (75.7)	
Pupil reactivity—no. of patients (%)			< 0.001
Both intact pupil reflex	166 (89.2)	15 (40.5)	
One unreactive pupil	4 (2.2)	3 (8.1)	
Both unreactive pupil	16 (8.6)	19 (51.4)	
Aneurysm location—no. of patients (%)			0.015
Anterior communicating artery	51 (24.4)	11 (29.7)	
Anterior cerebral artery and distal	13 (7.0)	2 (5.4)	
Middle cerebral artery and distal	51 (27.4)	5 (13.5)	
Internal carotid artery	11 (5.9)	9 (24.3)	
Posterior communicating artery	24 (12.9)	3 (8.1)	
Posterior circulation	22 (11.8)	2 (5.4)	
No aneurysm	10 (5.4)	4 (10.8)	
Unknown	4 (2.2)	1 (2.7)	
Hydrocephalus—no. of patients (%)	85 (45.7)	26 (70.3)	0.011
Intraventricular hemorrhage—no. of patients (%)	54 (29.0)	28 (75.7)	< 0.001

*SD* standard deviation, *BMI* body mass index, *TIA* transient ischemic attack, *CT* computed tomography

**Table 2:** Treatment characteristics

	Favorable neurological outcome (*n* = 186)	Poor neurological outcome (*n* = 37)	*p* value
Aneurysm treatment and timing—no. of patients (%)			< 0.001
Early treatment within 72 h	174 (93.5)	24 (64.9)	
Early but non-aneurysm detection	8 (4.3)	0 (0)	
Late treatment	1 (0.5)	1 (2.7)	
No treatment	3 (1.6)	12 (32.4)	
Aneurysm management—no. of patients (%)			
Coiling	108 (58.1)	16 (43.2)	0.140
Clipping	66 (35.5)	9 (24.3)	0.262
Endotracheal intubation during over 24 h	38 (20.4%)	33 (89.2%)	< 0.001
External ventricular drainage—no. of patients (%)	64 (34.4)	16 (43.2)	0.403
Delayed cerebral ischemia—no. of patients (%)	39 (21.0)	7 (18.9)	0.953
Decompressive craniectomy—no. of patients (%)	12 (6.5)	8 (21.6)	0.008
Barbiturate coma therapy—no. of patients (%)	2 (1.1)	3 (8.1)	0.042

### Optic nerve sheath diameters and their modifications

**Fig. 3:**
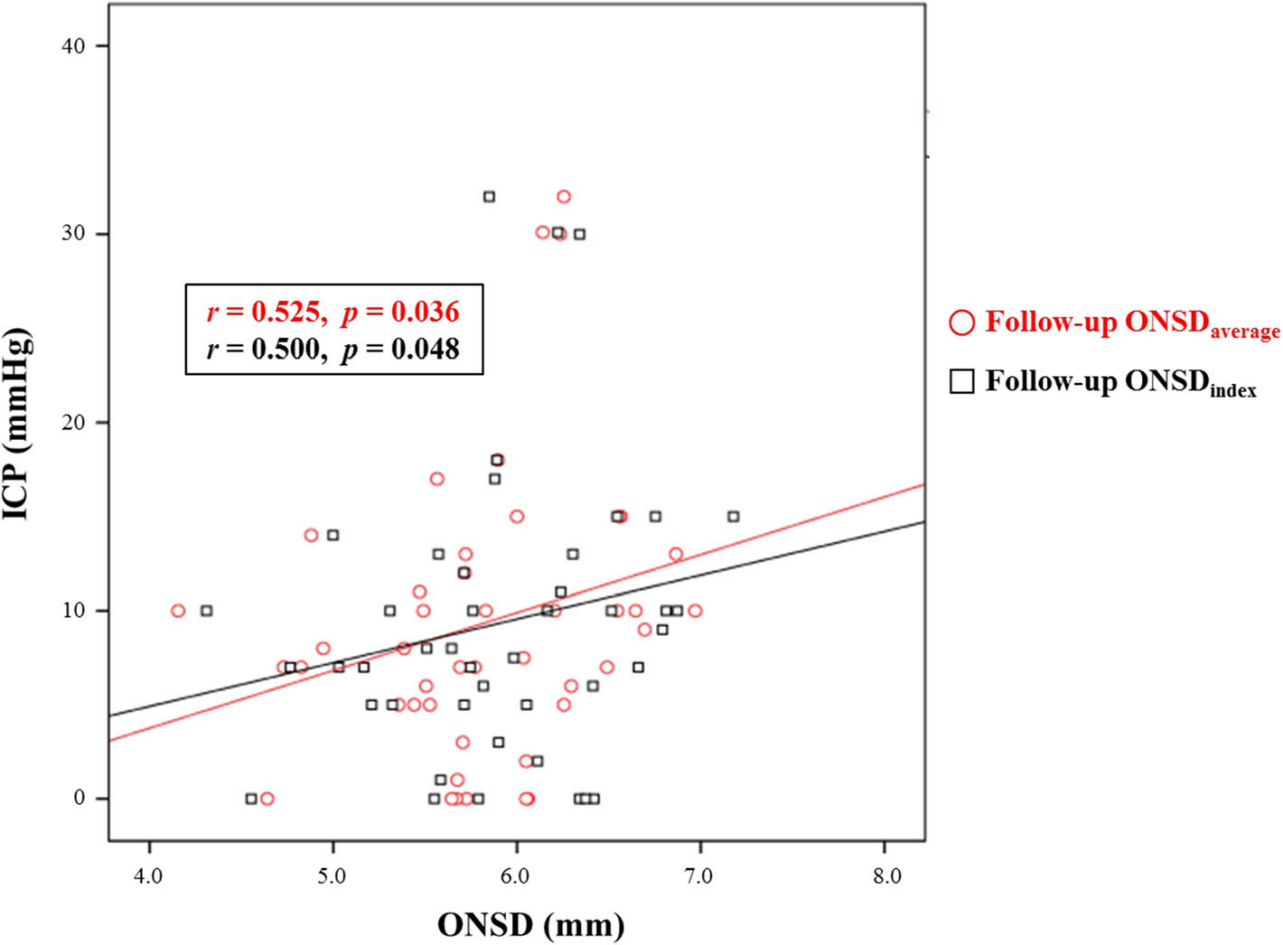
Scatter plot of intracranial pressure (ICP, mmHg) and optic nerve sheath diameter (ONSD, mm). Follow-up ONSD_average_ and follow-up ONSD_index_ were used in simple correlation analysis

**Fig. 4:**
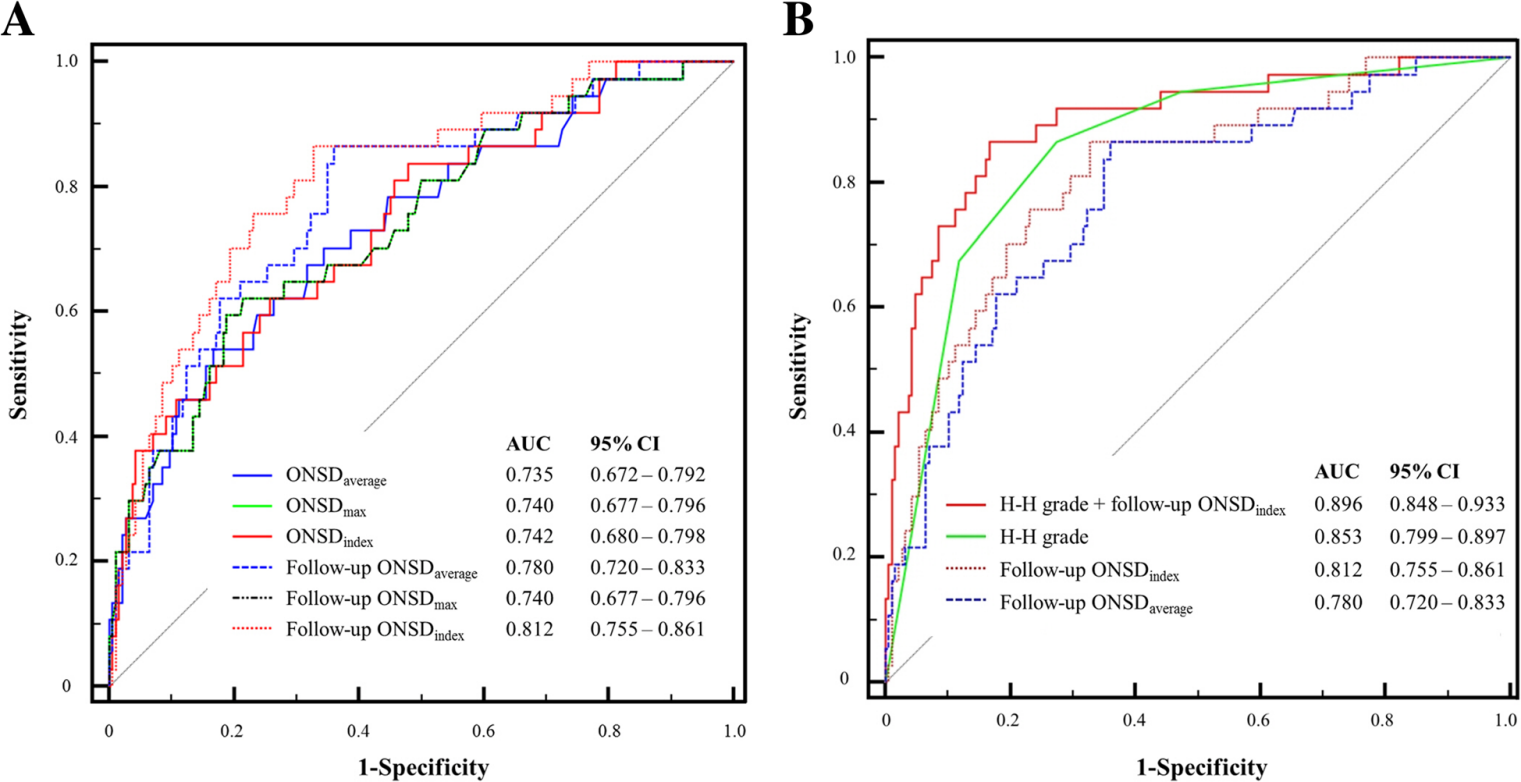
**a**, **b** Receiver operating characteristic curves for predicting poor outcomes using Hunt and Hess grade, the optic nerve sheath diameters, and their modifications (**a**). Although there were no differences between the areas under the curve (AUCs) of H-H grade, follow-up ONSD_average_, and follow-up ONSD_index_, the performance of a composite of H-H grade and follow-up ONSD_index_ was strongly associated with poor neurological outcomes compared with the use of either marker alone (all *p* < 0.006) (**b**). CI confidence interval, ONSD optic nerve sheath diameter, H-H grade Hunt and Hess grade

In this study, initial and follow-up ONSDs in the poor neurological outcome group were significantly greater than in the favorable outcome group (Table 3). However, initial ETD and follow-up ETD did not differ significantly between the favorable and poor neurological outcome groups (*P* = 0.504 and *P* = 0.295, respectively). In addition, changes in ONSD (∆ONSD_average_) between initial and follow-up CT were not associated with prognosis. Although invasive ICP monitoring was performed in 80 (35.9%) patients during hospitalization, the ICP was measured in only 21 (9.4%) patients at the time of follow-up CT. Based on simple correlation analysis, the ICP showed a linear correlation with follow-up ONSD_average_ and follow-up ONSD_index_ (*r* = 0.525, *P* = 0.036 and *r* = 0.500, *P* = 0.048, respectively) (Fig. 3). In ROC curve analysis for prediction of poor neurological outcomes (Fig. 4a), the AUCs of ONSD_index_ were greater than those of ONSD_average_ during the initial CT and follow-up CT. However, no significant differences existed between the predictive performance of ONSD_index_ and ONSD_average_ during the initial CT and follow-up CT (*P* = 0.764, *P* = 0.086, respectively). The performance of ONSD_index_ in follow-up CT was significantly better than that of ONSD_index_ and ONSD_average_ in initial CT (*P* = 0.033, *P* = 0.022, respectively). The *C*-statistic of follow-up ONSD_index_ was 0.812 (95% confidence interval (CI) 0.754 to 0.861). A cutoff > 6.22 had a sensitivity of 70.3% (95% CI 53.0 to 84.1%) and a specificity of 80.7% (95% CI 74.2 to 86.1%). The *C*-statistic of H-H grade was 0.853 (95% CI 0.799 to 0.897). A cutoff > 3 had a sensitivity of 86.5% (95% CI 71.2 to 95.5%) and a specificity of 72.6% (95% CI 65.6 to 78.9%). Although no significant differences existed between the AUCs of H-H grade, the follow-up ONSD_average_, and the follow-up ONSD_index_, the composite of H-H grade and the follow-up ONSD_index_ [AUC 0.896, Brier score 0.078, goodness-of-fit (Osius-Rojek) *Z* = − 0.5052, *p* = 0.613] showed the highest ability to correctly predict poor neurological outcomes rather than either marker alone (all *p* < 0.006, Fig. 4b). Based on our definitions of poor markers as H-H grade > 3 and follow-up ONSD_index_ > 6.22, 107 (48.0%) patients had one or more poor markers (sensitivity 91.9% and specificity 60.8%). Of these patients, 39 (17.5%) carried two poor markers (H-H grade > 3 [poor-grade SAH] and follow-up ONSD_index_ > 6.22, sensitivity 64.9%, and specificity 91.9%).

**Table 3:** The optic nerve sheath diameters and their modifications according to neurological outcomes

	Favorable neurological outcome (*n* = 186)	Poor neurological outcome (*n* = 37)	*p* value
ONSD_average_	5.86 ± 0.56	6.45 ± 0.71	< 0.001
ONSD_max_	5.99 ± 0.57	6.62 ± 0.75	< 0.001
ETD_average_	22.78 ± 1.18	22.64 ± 1.14	0.504
ONSD_index_	5.15 ± 0.52	5.70 ± 0.60	< 0.001
Follow-up ONSD_average_	5.77 ± 0.59	6.47 ± 0.71	< 0.001
Follow-up ONSD_max_	5.99 ± 0.57	6.62 ± 0.75	< 0.001
Follow-up ETD_average_	22.89 ± 1.65	22.65 ± 1.13	0.295
Follow-up ONSD_index_	5.06 ± 0.55	5.71 ± 0.53	< 0.001
∆ONSD_average_	0.01 ± 0.07	−0.01 ± 0.06	0.139

*ONSD* optic nerve sheath diameter, *ETD* eyeball transverse diameter, *∆ONSD*_*average*_ follow-up ONSD_average_ minus initial ONSD_average_

## Discussion

In this study, we evaluated whether ONSDs and their modifications could be used to predict neurological outcomes of patients with SAH. The major study findings were as follows: (1) Patients with poor-grade SAH manifested considerable survival rate (79.5%) and favorable neurological prognoses (61.4%); (2) simultaneous measurements of ONSDs with brain CT and ICP were moderately correlated; (3) during initial and follow-up CT, the ONSDs of patients in the poor neurological outcome group were significantly greater than in those of the favorable neurological outcome group, and these markers might predict neurological outcomes of SAH patients. In addition, follow-up ONSDs predicted prognosis better than the initial ONSDs; (4) the combined H-H grade and ONSD measured with CT was strongly associated with poor neurological outcomes than the use of either marker alone (H-H grade > 3 [poor-grade SAH] and follow-up ONSD_index_ > 6.22).

Although it is difficult to predict the outcomes following SAH, the level of consciousness at admission is associated with patient prognosis [1, 3, 7]. The widely accepted predictors include H-H grade [5], WFNS grade based on the level of consciousness assessed using the Glasgow coma scale [6, 7], and more recently, VASOGRADE-Red [20]. In particular, patients with poor-grade SAH historically showed high mortality and poor neurological outcomes [7]. However, in recent years, a considerable number of patients with poor-grade SAH treated aggressively with early coil embolization and supportive neurointensive care achieved favorable neurological outcomes and survival [3, 7]. Therefore, to accurately evaluate the prognosis of patients with poor-grade SAH, predictors other than the initial level of consciousness are needed.

In SAH patients, poor neurological outcome is usually secondary to early brain injury, rebleeding, or delayed cerebral ischemia [1, 8, 9]. Although rebleeding, cerebral vasospasm, and delayed cerebral ischemia may occur in patients with initially normal ICP, early brain injury within the first 72 h after the hemorrhage associated with acute consequences of SAH induced sudden intracranial hypertension [1, 9]. Severely increased ICP, one of the major mechanisms of early brain injury, leads to decreased cerebral perfusion pressure, cessation of cerebral blood flow, and ultimately global ischemia and edema [9, 10]. Early brain injury may increase the risk of developing delayed cerebral ischemia [9, 21]. Therefore, early monitoring of intracranial hypertension may facilitate the prediction of neurological outcomes in patients with SAH. In addition, it may be also used to identify a subgroup of SAH patients with uncontrolled refractory intracranial hypertension, who are at risk of death or delayed neurological impairment and benefit from different management strategies such as decompressive craniectomy or barbiturate coma therapy. Eventually, early monitoring of intracranial hypertension based on the measurement of ONSD may be useful to treat SAH patients.

ONSD has been proposed as an alternative parameter for detection of increased intracranial pressure [11]. The optic nerve is surrounded by cerebrospinal fluid because it is a part of the central nervous system. Therefore, increased ICP is transmitted through the subarachnoid space surrounding the optic nerve within the nerve sheath, especially the retrobulbar segment, unless circulation of cerebrospinal fluid is not blocked [11]. The ONSD measured during the initial brain CT may be correlated with neurologic outcomes after traumatic brain injury [12, 22]. Simultaneous measurement of ONSD with initial CT and intracranial pressure were correlated, and ONSD was indicative of intracranial hypertension in patients with severe traumatic brain injury [12, 22]. In this study, ONSD measured with CT was also a good predictive marker associated with ICP and prognosis in SAH patients.

Although ONSD is considered an indirect marker for ICP, the optimal cutoff for an abnormal ONSD indicating elevated ICP and its associated factors have been unclear [23]. In addition, there are limited reports investigating the role of ONSD modifications or indices in detecting intracranial hypertension compared with absolute ONSD [14, 15]. The ONSD correlates strongly with ETD in healthy people, and the ONSD/ETD ratio may provide highly reliable data than the absolute ONSD as a marker of ICP [23]. However, in this study, there were no significant differences between the ONSD/ETD ratio and absolute ONSD in predicting poor neurological outcomes. Therefore, future studies with larger cohorts are needed to confirm these findings.

In this study, we evaluated the potential usefulness of ONSD in detecting intracranial hypertension non-invasively and rapidly and determined its value in clinical decision-making for the management of SAH patients. ONSD has been proposed as an indirect and alternative parameter for detection of intracranial hypertension [11, 12]. However, ICP alone is not the only determinant of poor neurological outcome. Therefore, ONSD alone cannot be considered as the ideal predictor of neurological outcomes in SAH patients. Eventually, ONSD may be used in combination with clinical grading scales to improve prognostic accuracy.

This study has several limitations. First, it was a retrospective review of medical records. Therefore, the GOS was also retrospectively determined based on medical records. Second, the non-randomized nature of the registry data may have resulted in selection bias. Although brain CT scans were performed within 48 h after SAH, a major limitation of this study may be related to the CT scans performed at different times. Third, the brain CT scan was performed by two different scanners, and ONSDs were also obtained using two different commercial image-viewing software products, which might have resulted in bias. In addition, ONSD measurement requires high image resolution and specific CT settings such as optimal slice thickness and slice angle with the skull base. The heterogeneity of CT scan protocols may explain the relatively weak correlation between ONSD and ICP in this study [24]. Fourth, invasive ICP monitoring was performed in a limited number of patients after aneurysmal treatment. Finally, our study has limited statistical power due to the small sample size. Although it still provides valuable insight, prospective large-scale studies are needed to evaluate the usefulness of ONSD using brain CT in predicting neurological outcomes of patients with SAH to arrive at evidence-based conclusions.

## Conclusion

ONSDs measured with CT may be used to predict neurological outcomes of SAH patients. Furthermore, a composite parameter of H-H grade and ONSD may be helpful to more accurately assess the prognosis of SAH patients.

## Abbreviations

AUC: Area under the curve
CT: Computed tomography
ETD: Eyeball transverse diameter
GOS: Glasgow Outcome Scale
H-H: Hunt and Hess
ICP: Intracranial pressure
IQR: Interquartile range
ONSD: Optic nerve sheath diameter
ROC: Receiver operating characteristic
SAH: Subarachnoid hemorrhage

## References

[1] de Oliveira Manoel AL, Goffi A, Marotta TR, Schweizer TA, Abrahamson S, Macdonald RL (2016). The critical care management of poor-grade subarachnoid haemorrhage. Crit Care.

[2] Naval NS, Chang T, Caserta F, Kowalski RG, Carhuapoma JR, Tamargo RJ (2013). Improved aneurysmal subarachnoid hemorrhage outcomes: a comparison of 2 decades at an academic center. J Crit Care.

[3] Konczalla J, Seifert V, Beck J, Guresir E, Vatter H, Raabe A (2018). Outcome after Hunt and Hess grade V subarachnoid hemorrhage: a comparison of pre-coiling era (1980-1995) versus post-ISAT era (2005-2014). J Neurosurg.

[4] Wartenberg KE (2011). Critical care of poor-grade subarachnoid hemorrhage. Curr Opin Crit Care.

[5] Hunt WE, Hess RM (1968). Surgical risk as related to time of intervention in the repair of intracranial aneurysms. J Neurosurg.

[6] Teasdale GM, Drake CG, Hunt W, Kassell N, Sano K, Pertuiset B (1988). A universal subarachnoid hemorrhage scale: report of a committee of the World Federation of Neurosurgical Societies. J Neurol Neurosurg Psychiatry.

[7] Taylor CJ, Robertson F, Brealey D, O'Shea F, Stephen T, Brew S (2011). Outcome in poor grade subarachnoid hemorrhage patients treated with acute endovascular coiling of aneurysms and aggressive intensive care. Neurocrit Care.

[8] Rowland MJ, Hadjipavlou G, Kelly M, Westbrook J, Pattinson KT (2012). Delayed cerebral ischaemia after subarachnoid haemorrhage: looking beyond vasospasm. Br J Anaesth.

[9] Flynn L, Andrews P. Advances in the understanding of delayed cerebral ischaemia after aneurysmal subarachnoid haemorrhage. F1000Res. 2015;4. 10.12688/f1000research.6635.1. PMID: 26937276. 10.12688/f1000research.6635.1 PMC4752028 26937276

[10] Johnson U, Engquist H, Lewen A, Howells T, Nilsson P, Ronne-Engstrom E (2017). Increased risk of critical CBF levels in SAH patients with actual CPP below calculated optimal CPP. Acta Neurochir.

[11] Hwan Kim Y, Ho Lee J, Kun Hong C, Won Cho K, Hoon Yeo J, Ju Kang M (2014). Feasibility of optic nerve sheath diameter measured on initial brain computed tomography as an early neurologic outcome predictor after cardiac arrest. Acad Emerg Med.

[12] Sekhon MS, Griesdale DE, Robba C, McGlashan N, Needham E, Walland K (2014). Optic nerve sheath diameter on computed tomography is correlated with simultaneously measured intracranial pressure in patients with severe traumatic brain injury. Intensive Care Med.

[13] Legrand A, Jeanjean P, Delanghe F, Peltier J, Lecat B, Dupont H (2013). Estimation of optic nerve sheath diameter on an initial brain computed tomography scan can contribute prognostic information in traumatic brain injury patients. Crit Care.

[14] Vaiman M, Gottlieb P, Bekerman I (2014). Quantitative relations between the eyeball, the optic nerve, and the optic canal important for intracranial pressure monitoring. Head Face Med.

[15] Bekerman I, Sigal T, Kimiagar I, Ben Ely A, Vaiman M (2016). The quantitative evaluation of intracranial pressure by optic nerve sheath diameter/eye diameter CT measurement. Am J Emerg Med.

[16] DeLong ER, DeLong DM, Clarke-Pearson DL (1988). Comparing the areas under two or more correlated receiver operating characteristic curves: a nonparametric approach. Biometrics.

[17] Schisterman EF, Perkins NJ, Liu A, Bondell H (2005). Optimal cut-point and its corresponding Youden Index to discriminate individuals using pooled blood samples. Epidemiology.

[18] Ruopp MD, Perkins NJ, Whitcomb BW, Schisterman EF (2008). Youden Index and optimal cut-point estimated from observations affected by a lower limit of detection. Biom J.

[19] Chen P, Tebbs JM, Bilder CR (2009). Global goodness-of-fit tests for group testing regression models. Stat Med.

[20] de Oliveira Manoel AL, Jaja BN, Germans MR, Yan H, Qian W, Kouzmina E (2015). The VASOGRADE: a simple grading scale for prediction of delayed cerebral ischemia after subarachnoid hemorrhage. Stroke.

[21] Sabri M, Lass E, Macdonald RL (2013). Early brain injury: a common mechanism in subarachnoid hemorrhage and global cerebral ischemia. Stroke Res Treat.

[22] Sekhon MS, McBeth P, Zou J, Qiao L, Kolmodin L, Henderson WR (2014). Association between optic nerve sheath diameter and mortality in patients with severe traumatic brain injury. Neurocrit Care.

[23] Kim DH, Jun JS, Kim R (2017). Ultrasonographic measurement of the optic nerve sheath diameter and its association with eyeball transverse diameter in 585 healthy volunteers. Sci Rep.

[24] Monnin P, Sfameni N, Gianoli A, Ding S (2017). Optimal slice thickness for object detection with longitudinal partial volume effects in computed tomography. J Appl Clin Med Phys.

